# Factors affecting the outcomes after bidirectional Glenn shunt: two decades of experience from a tertiary referral center

**DOI:** 10.1186/s43044-023-00381-2

**Published:** 2023-06-28

**Authors:** Aly A. Yousef, Ahmed F. Elmahrouk, Tamer E. Hamouda, Abdelmonem M. Helal, Ahmed M. Dohain, Abdulhadi Alama, Mohammad S. Shihata, Osman O. Al-Radi, Ahmed A. Jamjoom, Mohamed H. Mashali

**Affiliations:** 1 Division of Pediatric Critical Care, Department of Pediatrics, King Faisal Specialist Hospital and Research Center, Jeddah, Saudi Arabia; 2 Department of Pediatrics, Faculty of Medicine, Helwan University, Cairo, Egypt; 3 Division of Cardiac Surgery, Cardiovascular Department, King Faisal Specialist Hospital and Research Center, MBC J-16, P.O. Box:40047, Jeddah, 21499 Saudi Arabia; 4 Cardiothoracic Surgery Department, Tanta University, Tanta, Egypt; 5 Cardiothoracic Surgery Department, Benha University, Benha, Egypt; 6 Department of Pediatrics, Pediatric Cardiology Division, Cairo University, Cairo, Egypt; 7 Pediatric Cardiology Department, King Faisal Specialist Hospital and Research Center, Jeddah, Saudi Arabia; 8 Pediatric Cardiology Department, King Abdulaziz University, Jeddah, Saudi Arabia; 9 Cardiac Surgery Section, Department of Surgery, King Abdulaziz University, Jeddah, Saudi Arabia

**Keywords:** Bidirectional Glenn shunt, Single ventricle, Nakata index, Fontan palliation

## Abstract

**Background:**

Despite the improved management of patients with a single ventricle, the long-term outcomes are not optimal. We reported the outcomes of the bidirectional Glenn procedure (BDG) and factors affecting the length of hospital stay, operative mortality, and Nakata index before Fontan completion.

**Results:**

This retrospective study included 259 patients who underwent BDG shunt from 2002 to 2020. The primary study outcomes were operative mortality, duration of hospital stay, and Nakata index before Fontan. Mortality occurred in 10 patients after BDG shunt (3.86%). By univariable logistic regression analysis, postoperative mortality after BDG shunt was associated with high preoperative mean pulmonary artery pressure (OR: 1.06 (95% CI 1.01–1.23); *P* = 0.02). The median duration of hospital stay after BDG shunt was 12 (9–19) days. Multivariable analysis indicated that Norwood palliation before BDG shunt was significantly associated with prolonged hospital stay (β: 0.53 (95% CI 0.12–0.95), *P* = 0.01). Fontan completion was performed in 144 patients (50.03%), and the pre-Fontan Nataka index was 173 (130.92–225.34) mm^2^/m^2^. Norwood palliation (β: − 0.61 (95% CI 62.63–20.18), *P* = 0.003) and preoperative saturation (β: − 2.38 (95% CI − 4.49–0.26), *P* = 0.03) were inversely associated with pre-Fontan Nakata index in patients who had Fontan completion.

**Conclusions:**

BDG had a low mortality rate. Pulmonary artery pressure, Norwood palliation, cardiopulmonary bypass time, and pre-BDG shunt saturation were key factors associated with post-BDG outcomes in our series.

## Background

Patients with single-ventricle pathology are managed with a series of staged palliative procedures, including modified Blalock-Taussig shunt (MBTS), bidirectional Glenn shunt (BDG), and later Fontan completion [1]. Despite the improved management of patients with a single ventricle, the long-term outcomes are not optimal [2]. BDG shunt is used to unload the systemic ventricle and improve systemic oxygenation in patients with single ventricle physiology [3]; consequently, the outcomes of Fontan completion improved with the staged repair. BDG shunt can be performed as a step before Fontan completion or as part of a one-and-a-half ventricle repair [4, 5].

Several factors could affect mortality and pulmonary artery growth after BDG shunt, including age, diagnosis, and the dominant ventricle [6]. The effect of pulmonary artery growth and the Nakata index on the outcomes of Fontan completion is controversial; however, it was reported to be an independent predictor of the functional status after Fontan [7, 8]. Therefore, we reviewed our experience with patients who underwent BDG shunt and reported the outcomes of this staged palliative procedure and factors affecting the length of hospital stay, operative mortality, and Nakata index before Fontan completion.

## Methods

### Design and research ethics

We performed a retrospective study on 259 patients who underwent BDG shunt from 2002 to 2020 in our centre. The decision to perform the BDG shunt was based on the multidisciplinary meeting of our paediatrics heart team, including cardiologists, cardiothoracic surgeons, and intensivists.

The study was approved by the local institutional review board, and the need for parents/guardians' consent to participate was waived.

### Study data

Data were collected from papers and electronic charts. Preoperative data included age, weight, and height at the time of the procedure, diagnosis, and previous intervention. Echocardiographic data were collected from the most recent echocardiography before the intervention. Data identified from echocardiography included the dominant ventricle and its function and the atrioventricular valve (AVV) regurgitation and its degree (severe, moderate, mild, or absent). The patients who had cardiac catheterization before the BDG shunt were reviewed. Data extracted after reviewing catheterization images included oxygen saturation, mean pulmonary artery (PA) pressure, indexed pulmonary vascular resistance (PVRI), and ventricular end‐diastolic pressure (VEDP). The diameters of the branch pulmonary arteries were measured, and the Nakata index was calculated [6].

BDG shunt was performed on all patients with an end-to-side anastomosis on cardiopulmonary bypass (CPB). In patients with bilateral superior vena cava (SVC), a bilateral BDG shunt was performed. We collected data related to the type of BDG shunt, CPB and cross-clamp times, and concomitant procedures.

Postoperative hospital outcomes included the duration of mechanical ventilation, intensive care unit (ICU) and hospital stay, time of sternal closure, chylothorax, diaphragmatic paralysis, and hospital mortality. Postoperative saturation, the need for inhaled nitric oxide (INO), and sildenafil also were reported.

Patients were followed to Fontan completion, and the interstage mortality, Nakata index before Fontan, and post-Fontan mortality were reported.

Study outcomes were hospital mortality, duration of hospital stay, and Nakata index before Fontan.

### Statistical analysis

Descriptive analysis was used to present our data. Continuous data were presented according to the normality distribution using both mean and standard deviation or median and 25th–75th percentiles. Nominal data were presented as numbers and percentages. A comparison of the pre and postoperative oxygen saturation was performed using the paired t-test. Univariable logistic regression analysis was used to evaluate factors associated with hospital mortality. Multivariable negative binomial regression was used to study factors associated with the duration of hospital stay, and quantile regression was used to study factors associated with Nakata index pre-Fontan. Variables with a *P*-value of 0.15 or less in the univariable analysis were included in the multivariable model. Stata 16 (Stata Corp-College Station-TX-USA) was used for analysis.

## Results

### Patients' characteristics

The study included 259 patients who underwent BDG shunt. Their median age was 9 months (5.7–15 months), and the most common diagnoses were tricuspid atresia (TA) (*n* = 49, 18.99%), hypoplastic left heart syndrome (HLHS) (*n* = 46, 17.83%), and common atrioventricular canal (CAVC) (*n* = 39, 15.12%). The left ventricle was dominant in 94 patients (36.29%), and the dominant right ventricle was found in 89 patients (34.36%).

BDG shunt was the first stage in 102 patients (39.69%) and was preceded by Norwood in 69 patients (26.85%), patent ductus arteriosus (PDA) stenting in 36 patients (14.01%), and MBTS in 30 patients (11.67%).

Most patients had normal preoperative ventricular function (*n* = 237, 95.56%). The atrioventricular valve was competent in 102 patients (43.40%), and 6 had severe regurgitation (2.55%). Preoperative saturation was 86% (80–91), and the mean pulmonary artery pressure was 14 (11–18) mmHg. (Table 1).

**Table 1:** Preoperative patients' characteristics

Preoperative data	
Age (months)	9 (5.7–15)
Weight (kg) (*n* = 258)	6.85 (5.6–8.45)
Height (cm) (*n* = 255)	67 (62–75)
Diagnosis (*n* = 258)	
Tricuspid atresia	49 (18.99%)
Hypoplastic left heart syndrome	46 (17.83%)
Common atrioventricular canal	39 (15.12%)
Other	36 (13.95%)
Transposition of great arteries	27 (10.47%)
Double outlet right ventricle	20 (7.75%)
Double inlet left ventricle	14 (5.43%)
Pulmonary atresia, Intact interventricular septum	14 (5.43%)
Mitral atresia	8 (3.10%)
Shon's complex	5 (1.94%)
Dominant ventricle	
Indeterminate	76 (29.34%)
Left ventricle	94 (36.29%)
Right ventricle	89 (34.36%)
Pre Glenn-intervention (*n* = 257)	
No intervention	102 (39.69%)
Norwood	69 (26.85%)
Patent ductus arteriosus stent	36 (14.01%)
Modified Blalock Taussig shunt	30 (11.67%)
Pulmonary artery banding	12 (4.67%)
Other	4 (1.56%)
Balloon atrial septostomy	3 (1.17%)
Right ventricular outflow tract stent	1 (0.39%)
Preoperative echo systemic ventricle function (*n* = 248)	
Normal	237 (95.56%)
Fair	5 (2.02%)
Depressed	6 (2.42%)
Atrioventricular valve regurgitation (*n* = 235)	
Normal	102 (43.40%)
Trace	17 (7.23%)
Mild	75 (31.91%)
Moderate	35 (14.89%)
Severe	6 (2.55%)
Preoperative saturation (%) (n-243)	86 (80–91)
Preoperative cardiac catheterization (*n* = 257)	203 (78.99%)
Mean pulmonary artery pressure (mmHg) (*n* = 148)	14 (11–18)
Systemic ventricle end-diastolic pressure (mmHg) (*n* = 155)	10 (7–13)
Qp/Qs room air (*n* = 160)	1 (0.68–1.58)
Indexed pulmonary vascular resistance room air (Woods unit) (*n* = 144)	1.49 (1–2.3)

Continuous data were presented as median and (25th–75th percentiles) and categorical data as numbers and percentages

### Operative data

Concomitant pulmonary artery plasty was performed in 67 patients (26.27%), atrial septectomy in 81 patients (31.64%), atrioventricular valve repair in 13 patients (5.06%), and repair of total anomalous pulmonary venous return in 9 patients (3.5%). The median cardiopulmonary bypass time was 55 (40–74) minutes, and the cross-clamp time was 10 (0–31) minutes (Table 2).

**Table 2:** Operative data

Type of Glenn (*n* = 257)	
1 Right BDG	195 (75.88%)
2 Bilateral BDG	46 (17.90%)
3 Left BDG	11 (4.28%)
4 Kawashima	5 (1.95%)
Pulmonary artery plasty (*n* = 255)	67 (26.27%)
Septectomy (n-256)	81 (31.64%)
Atrioventricular valve repair (*n* = 257)	13 (5.06%)
Total anomalous pulmonary venous return repair (*n* = 257)	9 (3.5%)
Cardiopulmonary bypass time (min) (*n* = 255)	55 (40–74)
Cross-clamp time (min) (*n* = 180)	10 (0–31)

BDG Bidirectional Glenn

Continuous data were presented as median and (25^th^–75th percentiles) and categorical data as numbers and percentages

### Postoperative outcomes

Early postoperative superior vena cava (SVC) pressure was 18 mmHg (15–20). Nitric oxide was used in 29 patients (11.28%), and sildenafil in 54 patients (21.09%). Postoperative oxygen saturation was 89% (85–93), which was significantly higher than the preoperative level (*P* ˂ 0.001). Sternal closure was delayed in 14 patients (5.45%). Chylothorax occurred in 32 patients (12.65%), phrenic nerve palsy in 26 patients (10.20%), and hospital mortality in 10 (3.86%) patients. The median duration of ICU stay was 5 (3–8) days, and hospital stay was 12 (9–19) days.

Interstage mortality occurred in 4 (1.54%) patients, and 144 (50.03%) had Fontan completion. Nakata index before Fontan was 173 (130.92–225.34). Mortality after Fontan was reported in 5 (1.93%) patients (Table 3).

**Table 3:** Postoperative outcomes

Early postoperative superior vena cava pressure (mmHg) (*n* = 153)	18 (15–20)
Inhaled nitric oxide (*n* = 257)	29 (11.28%)
Sildenafil (*n* = 256)	54 (21.09%)
Postoperative saturation (%) (*n* = 248)	89 (85–93)
Delayed sternal closure (*n* = 257)	14 (5.45%)
Early postoperative intervention (*n* = 169)	26 (15.38%)
Chylothorax (*n* = 253)	32 (12.65%)
Phrenic nerve palsy (*n* = 255)	26 (10.20%)
Mechanical ventilation (hours) (*n* = 246)	10 (6–23)
Chest tube duration (days) (*n* = 230)	6 (4–8)
ICU stay (days) (*n* = 170)	5 (3–8)
Hospital stay (days) (*n* = 253)	12 (9–19)
Fontan completion (*n* = 257)	98 (38.13%)
Hospital mortality	10 (3.86%)
Interstage mortality	4 (1.54%)
Mortality after Fontan	5 (1.93%)
Pre-Fontan data	
Pulmonary artery diameter (mm) (*n* = 86)	7.55 (6.3–8.8)
Left pulmonary artery cross sectional area (mm2) (*n* = 86)	44.77 (31.17–60.82)
Nakata index (mm2/m2) (*n* = 86)	173 (130.92–225.34)
Indexed right pulmonary artery diameter (mm/m2) (*n* = 86)	14.19 (12.14–16.41)
Indexed left pulmonary artery diameter (mm/m2) (*n* = 86)	11.88 (10–13.73)
Right/left pulmonary artery diameter (*n* = 86)	1.17 (1.03–1.38)

ICU Intensive care unit

Continuous data were presented as median and (25th–75th percentiles) and categorical data as numbers and percentages

### Factors associated with BDG shunt outcomes

By univariable logistic regression analysis, postoperative mortality after BDG shunt was associated with high preoperative mean pulmonary artery pressure (*P* = 0.02) (Table 4).

**Table 4:** Univariable analysis for factors affecting hospital mortality

	OR (95% CI)	p
Age	0.99 (0.97–1.02)	0.69
Weight	0.89 (0.70–1.14)	0.38
Height	0.98 (0.94–1.03)	0.45
Diagnosis	0.27 (0.61–1.15)	0.27
Dominant ventricle	0.46 (0.19–1.1)	0.08
Pre-Glenn intervention	0.74 (0.42–1.31)	0.30
Systemic ventricular function	1.27 (0.26–6.14)	0.76
Atrioventricular valve regurgitation	1.53 (0.88–2.65)	0.13
Pre Glenn saturation	0.97 (0.90–1.03)	0.34
Mean pulmonary artery pressure	1.06 (1.01–1.23)	0.02
Systemic ventricle end-diastolic pressure	1.04 (0.86–1.25)	0.68
Pulmonary wedge pressure	0.89 (0.63–1.27)	0.53
Qp/Qs	1.11 (0.48–2.55)	0.81
Pulmonary vascular resistance indexed (room air)	1.14 (0.54–2.4)	0.74
Type of bidirectional Glenn shunt	0.94 (0.34–2.60)	0.91
Pulmonary artery plasty	0.69 (0.14–3.35)	0.65
Septectomy	1.46 (0.40–5.33)	0.56
Atrioventricular valve repair	2.18 (0.25–18.60)	0.48
Cardiopulmonary bypass time	1.01 (0.99–1.03)	0.14
Cross-clamp time	1.01 (0.99–1.03)	0.16

Multivariable analysis indicated that Norwood palliation before BDG shunt was significantly associated with prolonged hospital stay (*P* = 0.01) (Table 5).

**Table 5:** Univariable and multivariable analysis of factors affecting hospital stay

	Univariable		Multivariable	
	β (95% CI)	P	β (95% CI)	P
Age (months)	− 0.004 (− 0.007–0.001)	0.003	0.01 (− 0.02–0.001)	0.09
Dominant right ventricle	0.21 (− 0.05–0.46)	0.12	− 0.09 (− 0.48–0.29)	0.64
Pre-Glenn intervention				
Norwood	0.58 (0.34–0.83)	< 0.001	0.53 (0.12–0.95)	0.01
MBTS	0.52 (0.20–0.85)	0.001	− 0.10 (− 0.56–0.34)	0.66
Pre-Glenn saturation	0.01 (− 0.0004–0.02)	0.06	0.02 (− 0.015–0.03)	0.5
Septectomy	− 0.20 (− 0.42–0.018)	0.07	0.02 (− 0.36–0.39)	0.92
CPB time (min)	0.005 (0.0009–0.008)	0.01	0.002 (− 0.003–0.007)	0.39

CPB: cardiopulmonary bypass; MBTS: modified Blalock Taussig shunt

Norwood palliation and preoperative saturation were inversely associated with the pre-Fontan Nakata index in patients who had Fontan completion (*P* = 0.003 and 0.03, respectively). (Table 6).

**Table 6:** Factors affecting pre-Fontan Nakata index

	Univariable		Multivariable	
	β (95% CI)	P	β (95% CI)	P
Age (months)	1.23 (− 0.16–2.62)	0.08	0.66 (− 0.40–1.7)	0.22
Pre-Glenn intervention				
Norwood	− 63.46 (− 106.88 to − 20.03)	0.01	− 0.61 (62.63–20.18)	0.003
MBTS	− 66.18 (− 119.86 to − 12.49)	0.02	− 44.68 (− 94.95–5.60)	0.08
Pre-Glenn saturation	− 2.44 (− 4.65 to − 0.22)	0.03	− 2.38 (− 4.49 to − 0.26)	0.03

MBTS: modified Blalock Taussig shunt

## Discussion

The staged approach for single ventricle pathology is associated with improved Fontan outcomes [9, 10]; however, the outcomes of Fontan completion are still not optimal. Improving the outcomes after each stage could impact the overall outcomes after Fontan. In this study, we reported our experience with BDG shunt over twenty years from our tertiary referral centers.

The study included 259 patients who underwent BDG shunt, and the primary outcomes of the study were operative mortality, duration of hospital stay, and the pre-Fontan Nakata index. Secondary outcomes included other postoperative complications, such as delayed sternal closure, chylothorax, diaphragmatic paralysis, Fontan completion, and interstage mortality.

The reported mortality in this series was 3.86%, and it was associated with higher pre-BDG shunt pulmonary artery pressure. The mortality rate reported after BDG shunt varies widely in the literature. Meyer and associates reported a 3.3% mortality rate among 61 children [11], and other series reported a mortality rate between 1 and 20% after BDG shunt [12–15].

Several factors could contribute to the wide variability of the reported mortality after BDG shunt. One of these factors is the use of cardiopulmonary bypass and the concomitant procedures performed. In our series, all patients had a repair on cardiopulmonary bypass, whether they had associated repair or not. Hussain and colleagues reported no mortality rate in 37 patients who had BDG without cardiopulmonary bypass [16]. Crotti and collaborators evaluated BDG shunt with and without cardiopulmonary bypass and found no difference in the outcomes between both approaches [17].

In our study, cardiopulmonary bypass has an effect on hospital stay but not on mortality. Another factor that could affect the outcomes of staged repair is concomitant atrioventricular valve regurgitation. Sinha and associates found that concomitant atrioventricular valve repair was associated with worse outcomes after the staged repair of single ventricles, but that was not the case in our study [18]. Similar to our series, Sethasathien, and associates found that high preoperative mean pulmonary artery pressure was an independent predictor of mortality after BDG shunt [15]. On the other hand, Silvilairat and colleagues found no effect of pulmonary artery pressure on mortality after BDG shunt [19]. Another study found that patients with accessory pulmonary blood flow had a trend of higher mortality and prolonged hospital stay after BDG shunt [20]. In concomitant to our study Talwar and associates found that younger age at BDG shunt was associated with prolonged hospital stay; however, age and pulmonary artery pressure did not affect mortality [21]. The previous Norwood palliation was another factor associated with a prolonged hospital stay in our series.

The effect of the Nakata index on the outcomes after Fontan is controversial. Adachi and coworkers found that the outcomes after Fontan, including the functional status, were not affected by the pulmonary artery index [22]. However, Ridderbos and colleagues found that the Nakata index was negatively associated with the follow-up duration and correlated with post-Fontan functional status [8]. Kansy and associates found that pre-BDG shunt pulmonary artery diameter was inversely correlated with the growth of the pulmonary artery [23]. Our series found that Norwood palliation and low pre-BDG oxygen saturation negatively correlated with the pre-Fontan Nakata index. Pulmonary artery growth after Norwood palliation is variable and is related to several factors, including the type of conduit [24, 25]. In clinical practice, we are sometimes deceived by high saturation, which could give us false image about pulmonary branch growth [26]. However, from our results here, we highly recommend that saturation alone is not a good indicator. The presence of collaterals to distal pulmonary artery branches could give high saturation and prevent pulmonary trees from growing.

Our study showed that BDG shunt using cardiopulmonary bypass has a low mortality rate. Several factors affecting the outcomes were studied, mainly related to the previous Norwood palliation and pre-BDG shunt pulmonary artery pressure. Patients at high risk of complications could benefit from a personalized approach to improve the outcomes.

Study limitations:

The study is limited by the retrospective design, and several unmeasured variables could have affected the outcomes. Additionally, the study had a wide group of patients, and the outcomes could be variables in those patients. We performed multivariable adjustments to account for these factors, while the number of patients who had mortality was low and multivariable adjustment for this outcome was not feasible. The study was performed over 20 years, and the practice and patient care could have changed during this time.

## Conclusions

BDG had a low mortality rate. Pulmonary artery pressure, Norwood palliation, cardiopulmonary bypass time, and pre-BDG shunt saturation were key factors associated with post-BDG outcomes in our series.

## Data Availability

Un-identified data are available upon request with the corresponding author.

## Abbreviations

BDG: Bidirectional Glenn procedure
MBTS: Modified Blalock-Taussig shunt
AVV: Atrioventricular valve
PA: Pulmonary artery
PVRI: Pressure, indexed pulmonary vascular resistance
VEDP: Ventricular end‐diastolic pressure
CPB: Cardiopulmonary bypass
SVC: Superior vena cava
ICU: Intensive care unit
INO: Inhaled nitric oxide
HLHS: Hypoplastic left heart syndrome
CAVC: Common atrioventricular canal
PDA: Patent ductus arteriosus
